# Short-Term Effects of Wearing Commercially and Non-commercially Available Motion Control Footwear Versus Standard Shoes on Running Biomechanics in Adults: A Systematic Review with Meta-analysis

**DOI:** 10.1186/s40798-025-00949-z

**Published:** 2025-11-21

**Authors:** Ali Esmaeili, AmirAli Jafarnezhadgero, Seyed Hamed Mousavi, Urs Granacher

**Affiliations:** 1 Department of Sport Biomechanics, Faculty of Educational Science and Psychology, University of Mohaghegh Ardabili, Ardabil, Iran; 2 Department of Sport Injuries and Biomechanics, Faculty of Sport Sciences and Health, University of Tehran, Tehran, Iran; 3 Department of Sport and Sport Science, Exercise and Human Movement Science, University of Freiburg, Freiburg, Germany

**Keywords:** Footwear, Pronation, Kinetics, Kinematics

## Abstract

**Background:**

There is controversy in the literature with regards to the short-term effects of wearing footwear with motion control features on running mechanics and whether commercially available footwear with motion control features has extra benefits compared with non-commercially available motion control footwear. In this systematic review with meta-analysis, we investigated the effects of wearing commercially available and non-commercially available footwear with motion control features versus standard shoes applied during one experimental session on lower limb joint angles and moments during running in adults.

**Methods:**

Five electronic databases (Scopus, PubMed, EMBASE, PEDro, Cochrane Central Register of Controlled Trials [CENTRAL]) were systematically searched for articles potentially eligible for inclusion from inception until September 2025. Footwear with motion control features were classified into commercially available motion control footwear without additional modifications (shoes with dual midsole material) versus non-commercially available footwear incorporating self-manufactured motion control features (shoes with heel flare or wedge). The main difference between these shoe types is how they control foot pronation. The control condition comprised standard (neutral) shoes. The outcome parameters were lower limb kinematics (e.g., peak rearfoot eversion) and kinetics (e.g., peak ankle inversion moment) during running. The modified version of the Downs and Black checklist was used to assess the methodological quality of the included studies. Within and between-group standardized mean differences (SMDs) with 95% confidence intervals (CI) were computed using a random-effects model to elucidate the effects of (i) wearing footwear with motion control features (both commercially available motion control shoes and non commercially available footwear with motion control features) compared to standard shoes (total effects) and (ii) commercially available motion control footwear without additional modifications versus non-commercially available footwear incorporating self-manufactured motion control features (subgroup analysis).

**Results:**

The systematic search revealed 11,623 hits and finally 18 studies were eligible for inclusion of which 14 were used for quantitative analyses. We observed significant total effects of wearing footwear with motion control features versus standard shoes during running on the peak rearfoot eversion angle (six studies; SMDs = − 0.87, 95% CI − 1.38 to − 0.35, *p* = 0.001, I^2^ = 66%) and the peak knee internal rotation angle (four studies; small SMDs = − 0.30, 95% CI − 2.58 to − 0.0, *p* = 0.05, I^2^ = 0%). The subgroup analyses revealed significantly lower peak rearfoot eversion in commercially available motion control footwear versus non-commercially available footwear incorporating self-manufactured motion control features (five studies SMDs = − 0.69, 95% CI − 1.19, − 0.18, *p* = 0.008, I^2^ = 50%). The included studies were rated as moderate methodological quality.

**Conclusions:**

This study revealed that wearing footwear with motion control features versus standard shoes has the potential to control rearfoot eversion and proximal segment motion in adults. The findings showed that wearing commercially available footwear with motion control features has extra benefits compared with non-commercially available motion control footwear. The observed findings for peak rearfoot eversion angle were statistically significant and clinically relevant. Nevertheless, more high-quality research is needed to elucidate the effects of footwear with motion control features application on running kinematics and kinetics as well as lower limb muscular activation.

**Supplementary Information:**

The online version contains supplementary material available at 10.1186/s40798-025-00949-z.

## Key Points

- The current systematic review with meta-analysis examined the (i) total short-term effects of wearing footwear with motion control features (both commercially available motion control footwear without additional modifications and non-commercially available footwear incorporating self-manufactured motion control features) versus standard shoes applied during one experimental test session on lower limb joint angles and moments during running in adults and (ii) the effects of commercially available motion control footwear versus non-commercially available footwear incorporating self-manufactured motion control features (subgroup analysis).
- This meta-analysis revealed that wearing footwear with motion control features versus standard shoes has the potential to control rearfoot eversion and proximal segment motion in adults.
- The findings showed that wearing commercially available footwear has extra benefits compared with non-commercially available motion control footwear.
- The observed findings on the overall effects of motion control shoes were statistically significant and clinically relevant for peak rearfoot eversion. Nevertheless, more high-quality research is needed to elucidate the effects of footwear with motion control features application on running mechanics as well as lower limb muscular activation.

**Supplementary Information:**

The online version contains supplementary material available at 10.1186/s40798-025-00949-z.

## Background

Footwear with motion control features (F-MCF) are defined as shoes with the goal to of limiting excessive foot pronation and improving lower limb alignment. For the purpose of this study, F-MCF was categorized as commercially available motion control footwear without additional modifications (CA-MCF) and non-commercially available footwear incorporating self-manufactured motion control features (NCA-MCF). The CA-MCF is commercially available in many footwear stores globally and is characterized by modified dual midsole material. Primarily for research purposes, NCA-MCF have been designed as footwear including specific motion control features such as medial posts, heel flares or wedges, and stiff heel counters with the goal to decrease excessive foot pronation, tibia and femur internal rotation, and frontal plane knee motion by modifying the deformation rates and geometry on the medial and lateral sides of the shoe [1]. By controlling excessive foot pronation, F-MCF may help to prevent lower limb injuries [2] and biomechanical tissue overload [3] associated with excessive overpronation.

The available original research on the effects of wearing F-MCF during one session on excessive foot pronation has produced conflicting results. While some studies indicated that F-MCF versus standard shoes can effectively manage excessive foot movement [4, 5], others could not find significant anti-pronation effects [6–9]. For instance, Clarke et al. [4] and Perry and Lafortune [5] suggested that F-MCF versus standard shoes can effectively control excessive foot pronation during running activities. McNair and Marshall [6] as well as other researchers [7–9] failed to demonstrate F-MCF related short-term effects on excessive foot pronation in runners. These authors used standard shoes as control conditions. Accordingly, the above mentioned studies cast doubt on the effectiveness of wearing F-MCF to reduce excessive overpronation.

Previously, attempts have been made to aggregate findings from original research in the form of systematic reviews and meta-analyses. For instance, Cheung and colleagues [10] included 14 studies in their meta-analysis and showed that the wearing F-MCF versus control condition (both standard shoes and barefoot running) during one session significantly reduced rearfoot eversion (mean difference = ~ 2°) and peak vertical impact (mean difference = ~ 9% of body mass). However, the meta-analysis could not find any significant effects of F-MCF on tibial internal rotation. In addition, Cheung et al.’s meta-analysis [10] has a some methodological drawbacks including their control condition (both standard shoes and barefoot running condition) and limited data availability for peak vertical impact force, tibial rotation, and foot pronation. In another meta-analysis, the same researchers examined the effects of F-MCF versus control condition (standard shoes and barefoot) during one session on foot pronation (i.e., rearfoot eversion) in healthy adults or individuals with pronated feet [11]. The results indicated that F-MCF improved rearfoot eversion control during running [10, 11].

Findings from previous meta-analyses [10, 11] indicate that there is still a void in the literature because these studies primarily focused on rearfoot motion and vertical impact peak, giving much less emphasis to the kinematics of the proximal joints (i.e., hip and knee joints). While previous work showed that F-MCF reduced rearfoot eversion, the consequence for the proximal segments was left uncertain because there was too few data. The current systematic review with meta-analysis builds on the existing literature base by including recently published data that examine other kinematic parameters, including knee internal rotation and ankle moments. For example, Hutchison et al., [12] were able to show significant reductions in both tibial and knee internal rotation during running when wearing F-MCF. These findings imply that the effects of F-MCF may not be limited to the ankle and foot but could also affect mechanical performance in more proximal joints. Unlike previous studies [10, 11], the current work utilized stricter inclusion criteria (i.e., F-MCF versus standard shoes), conducted subgroup analyses based on particular design characteristics (e.g., CA-MCF versus NCA-MCF), and carried out sensitivity analyses based on the quality of the included studies. Such methodological improvements, coupled with the incorporation of recent research, allowed for an in-depth analysis of multiple biomechanical parameters of the lower limbs and allowed for a richer explanation of the short-term effects of F-MCF. Given the limitations of previous systematic reviews with meta-analyses [10, 11] investigating the short-term effects of wearing F-MCF on running kinetics and kinematics in adults, it is timely to update and consolidate the available literature. Therefore, in this systematic review with meta-analysis we aimed to examine the (i) total short-term effects of wearing F-MCF (both CA-MCF and NCA-MCF) versus standard shoes applied during one experimental test session on lower limb joint angles and moments during running in adults and (ii) effects of CA-MCF versus NCA-MCF (subgroup analysis).

To improve clarity, the examined footwear has been categorized into two clusters: (1) CA-MCF; and (2) NCA-MCF. This categorization allows us to differ between complex but standardized, integrated designs and isolated single motion control features. In addition, the contrast will allow to determination of whether NCA-MCF has extra value compared with CA-MCF.

## Methods

This systematic review was conducted following the PERSiST guidelines for systematic reviews [13].

### Search Strategy

Five databases (Scopus, PubMed, EMBASE, PEDro, and Cochrane Central Register of Controlled Trials) were searched from inception until August 2025. Grey literature sources such as Science Direct, Clinicaltrial.gov, PROQUEST, and reference lists of identified articles were also systematically screened to identify additional eligible sources. The search strategy used for PubMed was adapted for each database (Appendix 1 - supplementary material). The search syntax followed the PICOS scheme and included MeSH terms and keywords combined with the Boolean operators AND, OR. Finally, the reference lists of already identified articles were screened for additional eligible papers.

### Eligibility Criteria

EndNote 20 software (Bld 14672, Clarivate, Philadelphia, PA, USA) was used for the systematic search and processing of potentially eligible articles. A PICOS (participants, intervention, comparator, outcomes, and study design) approach was applied to define inclusion and exclusion criteria (Table 1) a priori [14]. We included studies that examined individuals with either normal or pronated foot posture. Studies that were not foot-type specific were included if authors did not mention any foot pathology other than pronated feet. To be eligible for inclusion in this meta-analysis, articles had to be published in peer-reviewed journals in the English language. Articles not written in English were excluded (Table 1).

**Table 1:** Participants, intervention, comparator, outcomes, and study design (PICOS) framework for study inclusion and exclusion criteria

Category	Inclusion	Exclusion
Participants	Individuals aged ≥ 18 years with normal or pronated feet, or studies that did not specifically mention any foot pathology implying normal foot posture.	Individuals with adverse health events (e.g., injuries, recent surgery); individuals with neurological, systemic, or degenerative conditions; individuals aged < 18 years
Intervention	Commercially available motion control footwear without additional modifications and non-commercially available footwear incorporating self-manufactured motion control features within one experimental session	Other type of interventions or exposures such as foot orthoses or tape, long-term interventions
Comparator	Standard (neutral) shoes	Absence of a control condition, barefoot running
Outcomes	Lower limb kinematics (e.g., peak rearfoot eversion angle) and kinetics (e.g., peak knee adduction moment) during running	Measures of lower limb kinematics and kinetics during activities other than running (e.g., walking, jumping); no measures of lower limb kinematics and kinetics
Study design	Case control studies, case series, cohort study, cross sectional studies, baseline of randomized controlled trials	Case studies, systematic reviews and meta-analyses

### Study Selection

Two authors (A.E. and A.J.) reviewed the titles of potentially eligible studies based on the a priori defined inclusion and exclusion criteria. When the titles lacked sufficient information, the abstracts and full texts were examined to determine eligibility. In cases of disagreement between the two raters, a third author (S.H.M) was consulted to achieve consensus.

### Quality Assessment

The methodological quality of the included studies was evaluated separately by the same two authors (A.E., A.J.) using a modified 19-question Downs and Black checklist for non-randomized controlled trials [15]. This checklist includes eight reporting items (items 1, 2, 3, 4, 5, 6, 7, 10), two for external validity (items 11 and 12), five for internal validity (bias) (items 14, 15, 16, 18, 20), three for internal validity-confounding (items 21, 22, 25), and one for power (item 27). Scoring used “0” for “no” and “unable to determine”, and “1” for “yes” except for item 5 on principal confounders, which received “0” for “no”, “1” for “partially”, and “2” for “yes”. The overall quality score for each study was calculated as a percentage of the maximum possible score (20). Any discrepancies in scoring between the two authors were resolved through discussion with a third author (S.H.M). Studies were then categorized as high quality (75% or higher), moderate quality (60–74%), or low quality (60% or lower) [16].

### Data Collection

One author (A.E.) extracted the relevant data from the included studies into an Excel sheet. The extracted data included information on the study population (sex, age, and participants’ health status), the running protocol, type of F-MCF, applied control conditions and outcomes related to kinematic and kinetic data assessed during running. To minimize data extraction errors, all data were reviewed by the second author (A.J.). Kinematic data included peak joint angles, mean angles, and joint excursion values. Kinetic data comprised joint moments. If the respective data were not reported in the studies, we emailed the corresponding author or used a free web-based plot digitizer tool (https://plotdigitizer.com/webplotdigitizer-alternative) to extract data from graphs [17]. We initially aimed to perform subgroup analyses based on foot posture (normal vs. pronated feet). Given however that no outcome measure was reported in at least two studies per foot posture subgroup, it was impossible to calculate this contrast. Yet, we were able to compute subgroup analyses based on footwear design. Thus, we were able to contrast CA-MCF versus NCA-MCF. We categorized the footwear used in the included studies based on the methodological approach introduced in the meta-analysis by Cheung et al. [11]. Of note, outcomes that were reported in fewer than two studies were excluded from the meta-analysis. We compared movement and force variables for each joint between the conditions, F-MCF and standard shoes.

Each intervention shoe was classified as either CA-MCF or NCA-MCF. The CA-MCF included designs with midsole composition changes, such as dual-density midsoles. The NCA-MCF included wedged footwear or heel flare modifications.

### Statistical Analyses

The Cochrane Review Manager 5.1 (The Nordic Cochrane Centre, The Cochrane Collaboration, Copenhagen, Denmark) was used to compute and visually represent the synthesized quantitative data in the form of forest plots. Within and between-group standardized mean differences (SMDs) with 95% confidence intervals (CI) were computed using a random-effects model to elucidate the effects of (i) wearing F-MCF (both CA-MCF and NCA-MCF) compared to standard shoes (total effects) and (ii) CA-MCF versus NCA-MCF (subgroup analysis) on kinematic and kinetic variables during running. The SMDs were then categorized into trivial (0–0.2), small (0.2–5 moderate (0.5–0.8), and large (>0.8) [18–20]. Study heterogeneity was assessed using the I^2^ index. The level of heterogeneity was classified as high (>75%), moderate (50%–75%), and low (25–50%) [21].

### Assessment of Publication Bias

Publication bias and potential asymmetry of the effect sizes were visually assessed using a standard methodology funnel plot after reviewing the meta-analytical data [22] (see supplementary material in Appendix 2). The relationships between the computed effect sizes and the standard error were statistically verified using Egger’s statistics to determine whether the funnel plots were asymmetric [23]. In cases of asymmetry, the mean effect size obtained by adjusting the asymmetry was calculated using the trim-and-fill method and compared with the original average effect size to determine the number of potentially missing studies [24].

### Sensitivity Analysis

We performed sensitivity analyses to test the robustness of our results by excluding studies of “low quality” (score < 60%) according to the modified Downs and Black checklist [15]. Moreover, we performed heterogeneity-based sensitivity analysis, in which we excluded studies that were identified as primary contributors to heterogeneity in the analysis. To assess the potential impact of foot posture on our findings, we performed a sensitivity analysis by including studies only that explicitly reported information of participants with normal foot posture. For the sensitivity ananlysis, we excluded studies that recruited participants with pronated feet. This allowed us to isolate the effect of F-MCF in participants with normal feet. We then conducted the meta-analyses again for the primary outcomes and compared SMDs and p-values to the findings of the full statistical model.

## Results

### Study Selection

**Fig. 1:**
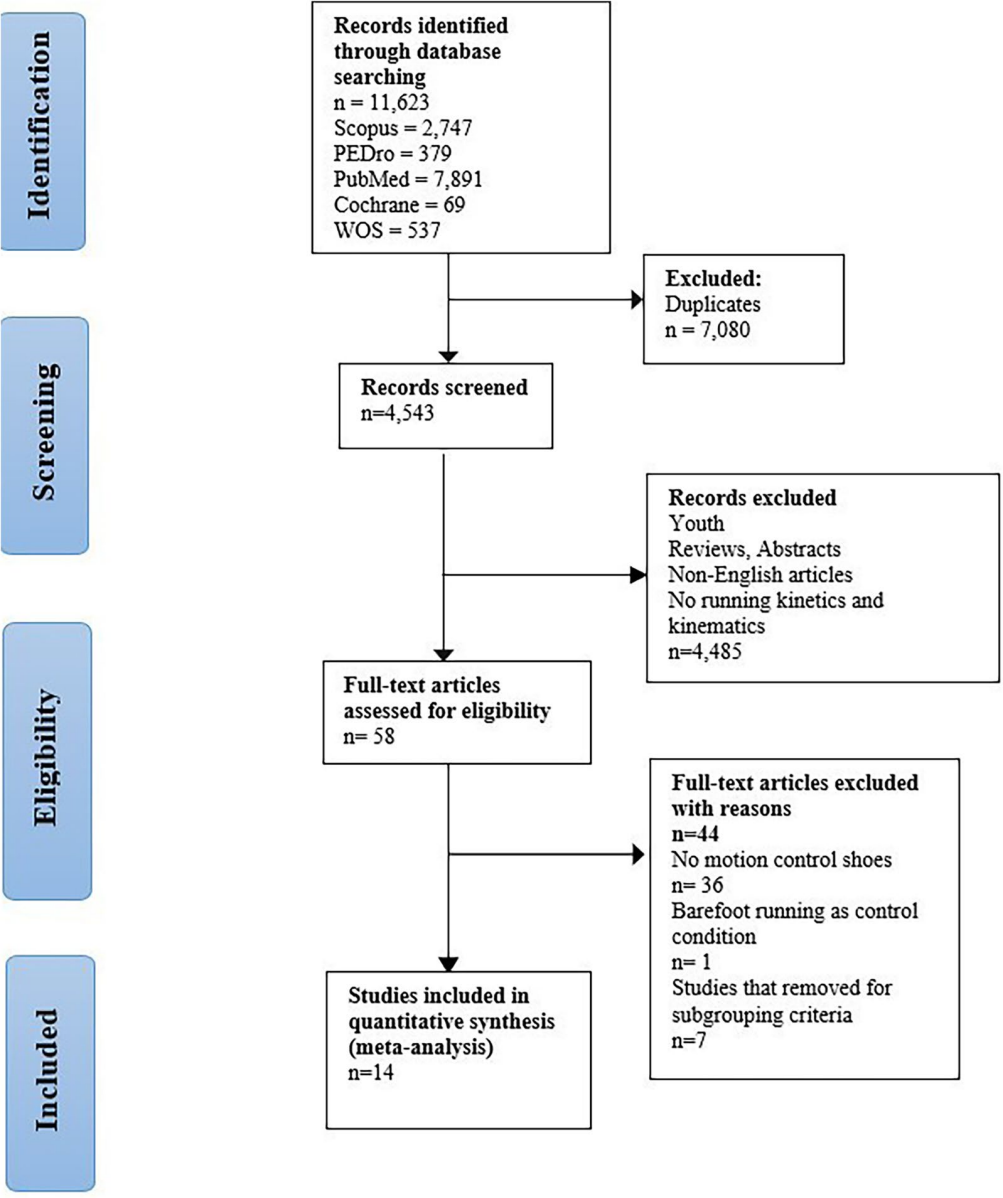
PRISMA flow diagram of studies included in this systematic review with meta-analysis

Our systematic search identified 11,623 studies. After eliminating duplicates, 4,543 studies remained. Following the screening of titles and abstracts, 58 full texts remained. Ultimately, 14 studies with 328 participants met the inclusion criteria for this systematic review with meta-analysis. Figure 1 displays a PRISMA flow chart depicting the study selection process.

### Study Characteristics

Table 2 shows the characteristics of the included studies. Kinematic data were reported in all 14 studies. Rearfoot kinematics were measured in ten studies [1, 4, 7, 25–31], knee kinetics in three studies [3, 32, 33], ankle kinematics in four studies [3, 32–34], and knee kinematics in five studies [3, 12, 26, 32, 33], hip kinematics in two studies [3, 32], ankle kinetics in two studies [3, 33], and finally hip kinetics in one study [3].

Study outcomes reported in a single study were excluded from the meta-analysis. Accordingly, the following studies were removed for the respective outcome parameters: peak ankle dorsiflexion [32], plantarflexion [32], abduction [32], peak ankle eversion [3], ankle abduction/adduction excursion [32], peak tibial rotation, tibial internal rotation excursion [25], tibial internal/external rotation excursion [35], peak knee abduction and abduction/adduction excursion [32], peak hip flexion, flexion/extension excursion, abduction/adduction excursion, and adduction angle [32], peak external rotation angle [3], and peak ankle, knee, and hip moment in three dimensions [3].

Twelve out of 14 studies used three-dimensional kinematic motion analysis, and nine studies applied force plates (either integrated into the treadmill or positioned in the center of a runway) to evaluate ground reaction forces while running. Running conditions also differed, with authors of some studies using treadmills and others overground running. Two studies applied two-dimensional kinematic motion analysis [28, 30]. All data used in the quantitative synthesis (e.g., peaks and standard deviations) were directly obtained from the original studies’ tables or the main texts. No data were extracted from graphs or figures using web-based digitizing tools. Butler et al., [25] reported values for participants with high and low arch feet. For this study, we only extracted data for the low arch foot group. The authors of five studies recruited participants with pronated feet [1, 3, 12, 25, 34]. Researchers from 11 studies investigated participants according to sex (males or females) [1, 3, 26, 27, 32–34], and scientists from three studies [25, 35, 36] examined mixed populations consisting of males and females. Scientists from another two studies did not report information on participants’ sex [4, 31].

Researchers from ten studies investigated participants according to sex (males, females) [1, 3, 7, 26, 28–30, 32–34], and scientists from two studies [12, 25] examined mixed populations consisting of males and females. Scientists from another two studies did not report information on participants’ sex [4, 31].

Ten studies [1, 3, 7, 12, 25, 26, 28, 31, 32, 34] tested CA-MCF. Three studies investigated the short-term effects of NCA-MCF [29, 30, 33]; one study examined the effects of both, CA-MCF and NCA-MCF [4]. Studies that examined the effects of NCA-MCF reported only rearfoot kinematics [29, 30, 33].

**Table 2:** Summary table of the 14 studies used for quantitative analyses

Study	*N*; sex; age (mean, SD)	Participants and foot condition	Footwear type (CA-MCF/NCAMCF)	Control condition	Running protocol	Specific outcomes	Kinematic and/or kinetic outcomes
Clarke et al. [4]	10 NR NR	Healthy runners	*A: CA-MCF; B: NCA-MC*F	Standard shoe (single midsole material/no wedge)	Treadmill running, 3.8 m/s	Rearfoot angles (maximum pronation, total range of rearfoot movement)	3D –
Nigg and Morlock [30]	14 Male NR	NR	*NCA-MCF*	Standard shoe (no wedge), adidas ZX 600	Individuals ran at 4 m/s along the 16 m runway	Total range of rearfoot movement	2D force plate
Hamill et al. [28]	8 female 20–26	Healthy	*CA-MCF*	Standard shoe (single midsole material) control shoe: only made of EVA	Treadmill running at 9.9–13.4 km/h	Rearfoot angles (maximum pronation, total range of rearfoot movement)	2D -
Milani et al. [29]	20 male 26 (3)	Healthy	*NCA-MC*F	Standard shoe (no wedge)	Individuals ran at 3.5 m/s along the runway	Maximum pronation	3D force plate
Van Gheluwe et al. [31]	30 NR 18–24	Healthy	*CA-MC*F	Standard shoe (single midsole material); nike air 180	Treadmill running at 3.8 m/s, at least three valid steps	Rearfoot angles (maximum pronation, total range of rearfoot movement)	3D –
Butler et al. [25]	20 Male and female 21.8 (3.2)	Arch height stratified: low arch:	*CA-MCF*	Standard shoes (new balance 1022NC)	Individuals ran at 3.57 m/s along a 25-m runway	Peak rearfoot eversion, eversion excursion, peak tibial internal rotation, and tibial internal rotation excursion; impact loading variables	3D force plate
Kersting and Brüggemann [7]	9 Male 28.9 (6.0)	Healthy experienced runners	*CA-MCF*	Standard shoes (single midsole material) asics gel 121 with a 35 shore value for the midsole.	The run-up distance toward the platform was 50 m, individuals ran at 4 m/s	Rearfoot angles (maximum pronation, total range of rearfoot movement)	3D force plate
Cheung et al. [1]	25 Female 23.6 (6.8)	Recreational runners with pronated feet	*CA-MC*F	Standard shoes (supernova cushion, Adidas)	25 Steps of the left foot; running at 2.78 m/s on treadmill	Rearfoot angle	3D -
Lilley et al. [26]	30 young females 21.2 (2.1)	Healthy	*CA-MC*F	Standard shoes (Adidas supernova glide)	Individuals ran at 3.5 m/s along the 10 m runway	Peak knee internal rotation and rearfoot eversion angles; knee adductor moment; peak loading rate of impact force	3D force plate
Hutchison et al. [12]	14 Male and female 22.3 (2.3)	Pronated feet	*CA-MCF*	Standard shoes (asics gel pulse 3)	Individuals ran along a 20 m runway	Peak knee flexion angle, peak knee internal rotation angle, total knee rotation ROM	3D force platform
Langley et al. [32]	28 Male 26 (7)	Healthy	*CA-MC*F	Standard shoes (asics gt 2000 2)	Participants ran at a self-selected pace (mean 2.9 m/s).	Hip, knee and ankle joint kinematics	3D
Jafarnezhadgero et al. [34]	22 Female 24.1 (5.6)	Recreational runners with pronated feet	*CA-MC*F	Standard shoes (asics women’s gel-nimbus 19)	Running at 3.3 m/s	Lower limb joint angles, moments	3D force plate
Jafarnezhadgero et al. [3]	26 female 24.1 (5.6)	Recreational runners with pronated feet	*CA-MC*F	Standard shoes (asics women’s gel-nimbus 19)	Running at 3.3 m/s	Peak negative free moments and peak ankle eversion angle during running	3D force plate
Weir et al. [33]	13 male 24.0 (4.4)	Healthy rearfoot recreational runners	*CA-MC*F	Standard shoes (brooks adrenaline gts-16)	Two 44-min prolonged treadmill running sessions at the preferred speed (mean 3.3 ± 0.4 m/s)	Knee and ankle joint moments and joint angle ROM	3D force plates implemented in treadmill

CA-MCF: commercially available motion control footwear; NCA-MCF: non-commercially available motion control footwear; ROM: range of motion, 2D: 2-dimensional; 3D: 3-dimensional; NR: not reported; SD: standard deviation

### Quality Assessment

The methodological quality of the included 14 studies amounted to 64.7% on the modified version of the Downs and Black checklist for non-randomized controlled trials [15]. This is indicative of a moderate level of quality (Table 3). Three studies were classified as high-quality [25, 26, 32], seven as moderate quality [1, 3, 7, 12, 28, 33, 34], and four were classified as low-quality [4, 29–31]. Only one study [33] involved assessors who were blind to the experimental condition (F-MCF or standard shoes) during the measurements; two studies [25, 32] reported a priori sample size computations (a priori power analysis).

**Table 3:** Downs and black methodological quality assessment score

Study	Reporting								External validity		Internal validity (bias)					Internal validity (confounding)			Power	Score (%)	Quality
	1	2	3	4	5	6	7	10	11	12	14	15	16	18	20	21	22	25	27		
Clarke et al. [4]	1	1	0	1	0	1	1	0	0	0	0	0	1	1	1	1	1	0	0	50	LQ
Nigg and Morlock [30]	1	1	0	1	0	1	1	0	0	0	0	0	1	1	1	1	0	0	0	45	LQ
Hamill et al. [28]	1	1	1	1	1	1	1	0	0	0	0	0	1	1	1	1	1	1	0	65	MQ
Milani et al. [29]	0	1	1	1	1	1	1	0	0	0	0	0	1	1	1	1	0	1	0	55	LQ
Van Gheluwe et al. [31]	1	1	0	1	0	1	1	0	0	0	0	0	1	1	1	1	0	0	0	45	LQ
Butler [25]	1	1	1	1	2	1	1	1	0	0	0	0	1	1	1	1	0	1	1	75	HQ
Kersting and Brüggemann [7]	1	1	1	1	2	1	1	1	0	0	0	0	1	1	1	1	0	1	0	70	MQ
Cheung [1]	1	1	1	1	2	1	1	1	0	0	0	0	1	1	1	1	0	1	0	70	MQ
Lilley [26]	1	1	1	1	2	1	1	0	1	0	0	0	1	1	1	1	0	1	0	70	MQ
Hutchison [12]	1	1	1	1	2	1	1	0	0	0	0	0	1	1	1	1	0	1	0	65	MQ
Langley [32]	1	1	1	1	1	1	1	1	0	0	0	0	1	1	1	1	1	1	1	75	HQ
Jafarnezhadgero et al. [34]	1	1	1	1	2	1	1	0	0	0	0	0	1	1	1	1	0	1	0	65	MQ
Jafarnezhadgero et al. [3]	1	1	1	1	2	1	1	0	1	0	0	0	1	1	1	1	0	1	0	70	MQ
Weir et al. [33]	1	1	1	1	1	1	1	1	0	0	1	0	1	1	1	1	0	1	0	70	MQ
Average score (mean [SD])																				64.68	MQ

1 = Yes; 0 = No; SD: Standard Deviation; HQ: High Quality (Score ≥ 75%); MQ: Moderate Quality (60% ≤ Score < 75%); LQ: Low Quality (Score < 60%)

### Short-Term Effects of Wearing F-MCF Versus Standard Shoes on Lower Limb Joint Angles

#### Rearfoot

**Fig. 2:**
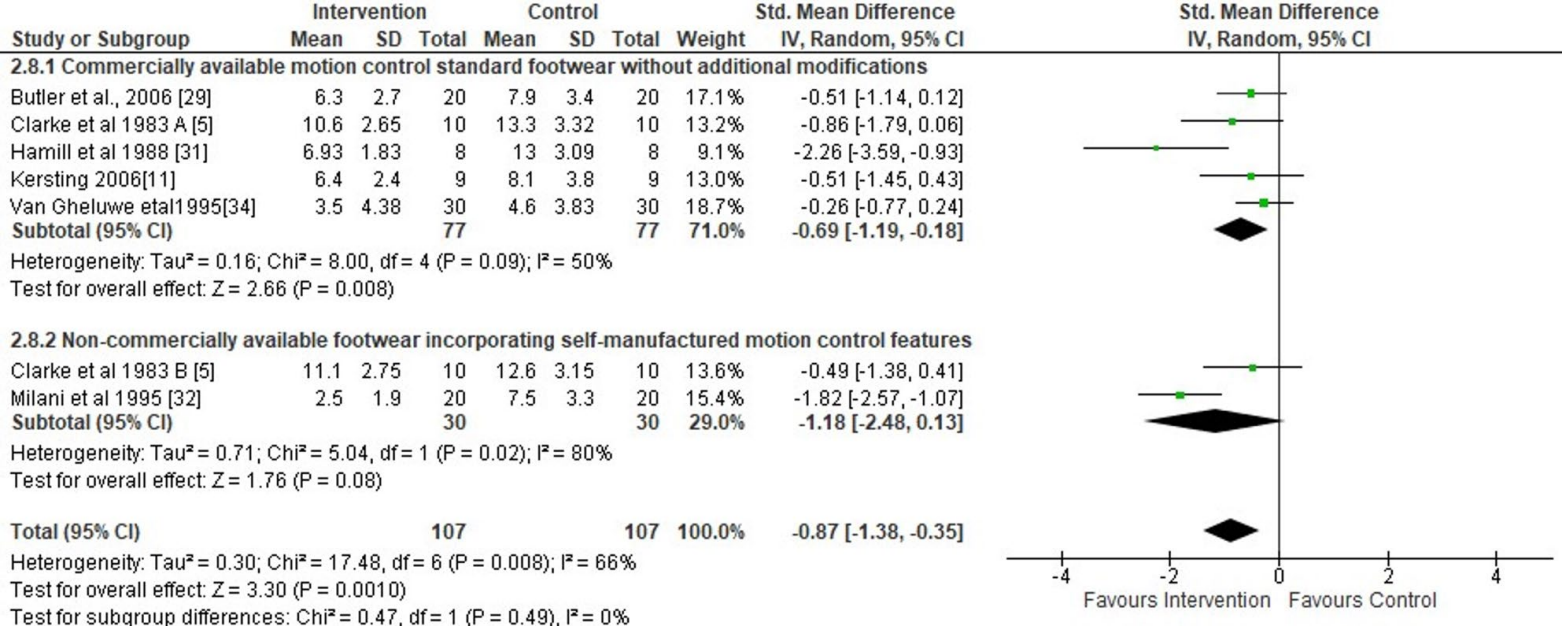
Forest plot illustrating the short-term effects of intervention (total short-term effects of wearing footwear with motion control features [both commercially available motion control footwear without additional modifications and non-commercially available footwear incorporating self-manufactured motion control features] and subgroup analysis of commercially available motion control footwear without additional modifications versus non-commercially available footwear incorporating self-manufactured motion control features) compared to standard shoes as control on peak rearfoot eversion angles during running. The subtotal effect of the different types of motion control shoes was calculated for each parameter and for the total effect as standardized mean differences (95% CI). SD: Standard deviation; Std: Standardized; CI: Confidence interval. **A** Commercially available motion control standard footwear without additional modifications; **B** Non-commercially available footwear incorporating self-manufactured motion control features

Researchers from six studies evaluated the short-term effects of F-MCF on peak rearfoot eversion [4, 7, 25, 28, 29, 31]. This meta-analysis showed significant effects of wearing F-MCF versus standard shoes during running on the peak rearfoot eversion angle (large SMDs=−0.87, 95% CI −1.38 to −0.35, *p* = 0.001) (Fig. 2). More specifically, across the six included studies, the peak rearfoot eversion angle was 2.85° (95% CI −4.36 to −1.34) lower in the F-MCF condition compared to the control (Fig. 2). The analysis further revealed a moderate level of study heterogeneity (I^2^ = 66%). The subgroup analyses of the type of F-MCF revealed significantly lower peak rearfoot eversion angles in the CA-MCF versus the NCA-MCF (five studies SMDs= −0.69, 95% CI −1.19, −0.18, *p* = 0.008, I^2^ = 50%) (Fig. 2). Egger’s statistics did not reveal statistically significant effects (*p* = 0.09). Thus, the findings of this meta-analysis were not flawed by publication bias.

**Fig. 3:**
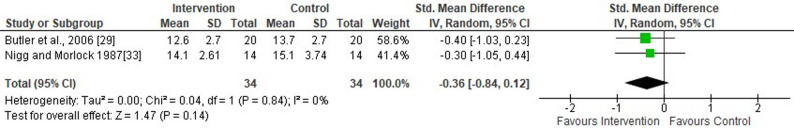
Forest plot illustrating the short-term effects of intervention (total short-term effects of wearing footwear with motion control features [both commercially available motion control footwear without additional modifications and non-commercially available footwear incorporating self-manufactured motion control features] and subgroup analysis of commercially available motion control footwear without additional modifications versus non-commercially available footwear incorporating self-manufactured motion control features) compared to standard shoes as control on rearfoot eversion excursion during running. The total effect was calculated as standardized mean differences (95% CI). SD: Standard deviation; Std: Standardized; CI: Confidence interval

The authors of two studies evaluated the short-term effects of wearing F-MCF versus standard shoes on rearfoot eversion excursion [25, 30]. No significant effects were found of wearing F-MCF versus standard shoes during running on rearfoot eversion excursion (Fig. 3). Egger’s test revealed no indication of publication bias (*p* = 0.83).

**Fig. 4:**
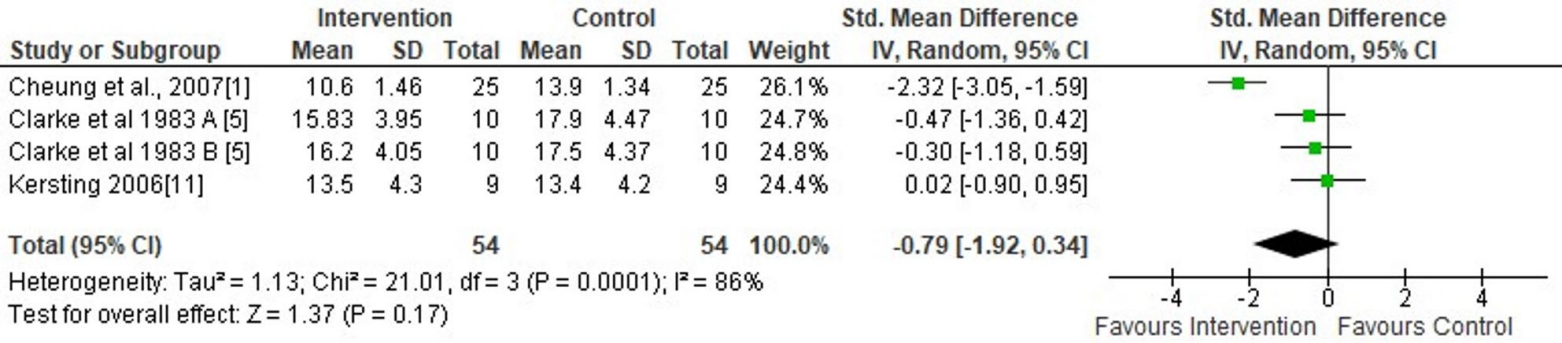
Forest plot illustrating the short-term effects of intervention [total short-term effects of wearing footwear with motion control features (both commercially available motion control footwear without additional modifications and non-commercially available footwear incorporating self-manufactured motion control features] and subgroup analysis of commercially available motion control footwear without additional modifications versus non-commercially available footwear incorporating self-manufactured motion control features) compared to standard shoes as control on rearfoot eversion/inversion excursion during running. The total effect was calculated as standardized mean differences (95% CI). SD: Standard deviation; Std: Standardized; CI: Confidence interval. **A** Commercially available motion control standard footwear without additional modifications; **B** Non-commercially available footwear incorporating self-manufactured motion control features

Researchers from three studies evaluated the short-term effects of wearing F-MCF on rearfoot eversion/inversion excursion [1, 4, 7]. This meta-analysis (wearing F-MCF footwear versus standard shoes) and subgroup analysis (CA-MCF versus NCA-MCF) showed no significant effects during running on rearfoot eversion/inversion excursion (moderate SMDs=−0.79, 95% CI −1.92 to 0.34, *p* = 0.17) (Fig. 4). The analysis further revealed a high level of heterogeneity (I^2^ = 86%) (Fig. 4). Egger’s regression test revealed strong evidence of small-study effects (*p* < 0.001). The trim-and-fill analysis imputed one missing study on the left side of the funnel plot, yielding an adjusted effect estimate of g = −1.05 (95% CI: −2.01 to −0.08) compared to the original estimate of g = −0.79 (95% CI: −1.85 to 0.27).

#### Ankle

**Fig. 5:**
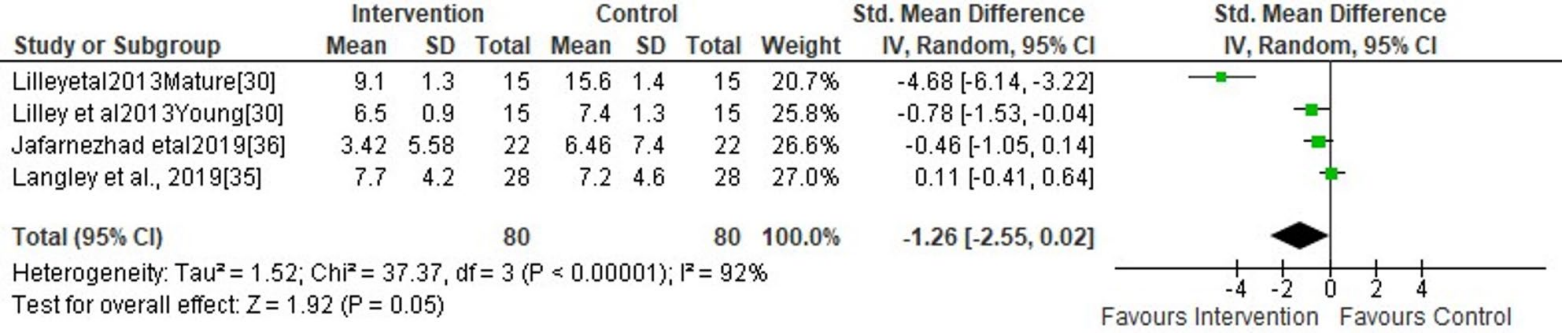
Forest plot illustrating the short-term effects of intervention (total short-term effects of wearing footwear with motion control features [both commercially available motion control footwear without additional modifications and non-commercially available footwear incorporating self-manufactured motion control features] and subgroup analysis of commercially available motion control footwear without additional modifications versus non-commercially available footwear incorporating self-manufactured motion control features) compared to standard shoes as control on peak ankle eversion during running. The total effect was calculated as standardized mean differences (95% CI). SD: Standard deviation; Std: Standardized; CI: Confidence interval. Mature: mature females, Young: young females

Three studies evaluated the effects of F-MCF application versus standard shoes on peak ankle eversion [26, 32, 34]. This meta-analysis showed no significant short-term effects of wearing F-MCF versus standard shoes during running on peak ankle eversion (small SMDs = −1.26, 95% CI −2.55 to 0.02, *p* = 0.22) (Fig. 5). The analysis further revealed a moderate level of heterogeneity (I2 = 53%) (Fig. 5). Egger’s regression test demonstrated highly significant evidence of small-study effects (*p* < 0.001). The trim-and-fill method did not identify any missing studies (g = −1.38, 95% CI: −3.43 to 0.67). This apparent contradiction suggests that while substantial asymmetry exists (per Egger’s test), the observed publication bias did not influence findings from the meta-analysis.

**Fig. 6:**
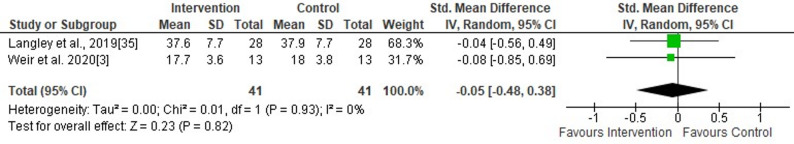
Forest plot illustrating the short-term effects of intervention [total short-term effects of wearing footwear with motion control features (both commercially available motion control footwear without additional modifications and non-commercially available footwear incorporating self-manufactured motion control features] and subgroup analysis of commercially available motion control footwear without additional modifications versus non-commercially available footwear incorporating self-manufactured motion control features) compared to standard shoes as control on ankle dorsiflexion/plantarflexion excursion during running. The total effect was calculated as standardized mean differences (95% CI). SD: Standard deviation; Std: Standardized; CI: Confidence interval

**Fig. 7:**
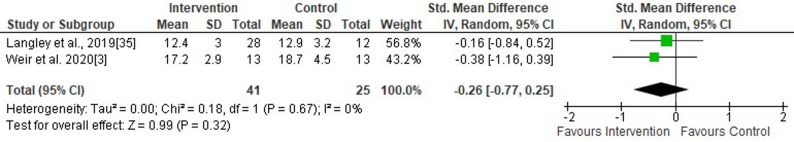
Forest plot illustrating the short-term effects of intervention [total short-term effects of wearing footwear with motion control features (both commercially available motion control footwear without additional modifications and non-commercially available footwear incorporating self-manufactured motion control features] and subgroup analysis of commercially available motion control footwear without additional modifications versus non-commercially available footwear incorporating self-manufactured motion control features) compared to standard shoes as control on ankle eversion/inversion excursion during running. The total effect was calculated as standardized mean differences (95% CI). SD: Standard deviation; Std: Standardized; CI: Confidence interval

Three studies evaluated the short-term effects of F-MCF application versus standard shoes on ankle dorsiflexion/plantarflexion and eversion/inversion excursion [32, 33]. This meta-analysis (wearing F-MCF versus standard shoes) and the respective subgroup analysis (CA-MCF subgroup versus NCA-MCF subgroup) did not show significant short-term effects during running on ankle dorsiflexion/plantarflexion and eversion/inversion excursion (Figs. 6 and 7). Egger’s test revealed no indication of publication bias for ankle dorsiflexion/plantarflexion excursion (*p* = 0.68) and ankle eversion/inversion excursion (*p* = 0.66).

#### Knee

**Fig. 8:**
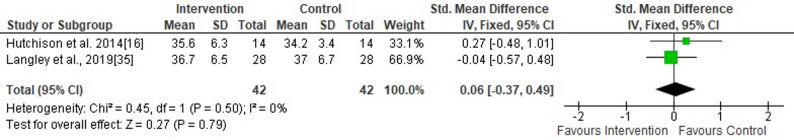
Forest plot illustrating the short-term effects of intervention [total short-term effects of wearing footwear with motion control features (both commercially available motion control footwear without additional modifications and non-commercially available footwear incorporating self-manufactured motion control features] and subgroup analysis of commercially available motion control footwear without additional modifications versus non-commercially available footwear incorporating self-manufactured motion control features) compared to standard shoes as control on peak knee flexion during running. The total effect was calculated as standardized mean differences (95% CI). SD: Standard deviation; Std: Standardized; CI: Confidence interval

**Fig. 9:**
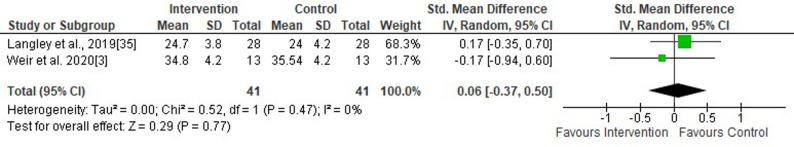
Forest plot illustrating the short-term effects of intervention [total short-term effects of wearing footwear with motion control features (both commercially available motion control footwear without additional modifications and non-commercially available footwear incorporating self-manufactured motion control features] and subgroup analysis of commercially available motion control footwear without additional modifications versus non-commercially available footwear incorporating self-manufactured motion control features) compared to standard shoes as control on knee flexion/extension excursion during running. The total effect was calculated as standardized mean differences (95% CI). SD: Standard deviation; Std: Standardized; CI: Confidence interval

**Fig. 10:**
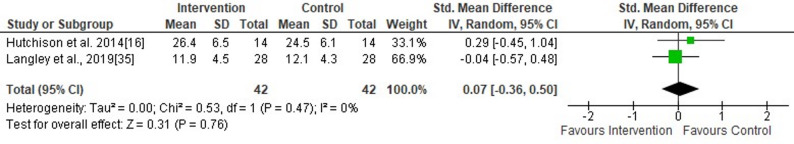
Forest plot illustrating the short-term effects of intervention (total short-term effects of wearing footwear with motion control features [both commercially available motion control footwear without additional modifications and non-commercially available footwear incorporating self-manufactured motion control features] and subgroup analysis of commercially available motion control footwear without additional modifications versus non-commercially available footwear incorporating self-manufactured motion control features) compared to standard shoes as control on knee internal/external rotation excursion during running. The total effect was calculated as standardized mean differences (95% CI). SD: Standard deviation; Std: Standardized; CI: Confidence interval

Two studies evaluated the short-term effects of MCF footwear application versus standard shoes on peak knee flexion [12, 32], knee flexion/extension excursion [32, 33], and internal/external rotation excursion [12, 32]. This meta-analysis (wearing F-MCF versus standard shoes) and the respective subgroup analysis (CA-MCF versus NCA-MCF) did not produce any significant effects during running on peak knee flexion, knee flexion/extension excursion, and internal/external rotation excursion (Figs. 8, 9 and 10). Egger’s test revealed no indication of publication bias for peak knee flexion (*p* = 0.48), knee flexion/extension excursion (*p* = 0.45), and internal/external rotation excursion (*p* = 0.45).

**Fig. 11:**
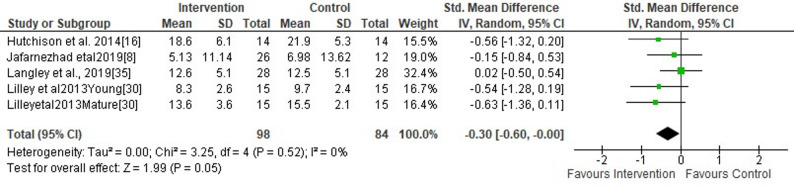
Forest plot illustrating the short-term effects of intervention [total short-term effects of wearing footwear with motion control features (both commercially available motion control footwear without additional modifications and non-commercially available footwear incorporating self-manufactured motion control features] and subgroup analysis of commercially available motion control footwear without additional modifications versus non-commercially available footwear incorporating self-manufactured motion control features) compared to standard shoes as control on peak knee internal rotation angle during running. The total effect was calculated as standardized mean differences (95% CI). SD: Standard deviation; Std: Standardized; CI: Confidence interval. Mature: mature females, Young: young females

Researchers from four studies evaluated the short-term effects of wearing F-MCF versus standard shoes on peak knee internal rotation angle [3, 12, 26, 32]. The findings indicated significant effects of wearing F-MCF versus standard shoes during running on peak knee internal rotation angle (small SMDs = −0.30, 95% CI −2.58 to −0.0, *p* = 0.05). More specifically, the peak knee internal rotation angle was 1.42° (95% CI −2.58 to −0.26) lower in the F-MCF condition compared to standard shoes (Fig. 11). The analysis further revealed no study heterogeneity with I^2^ = 0%. In addition, Egger’s test revealed no indication of publication bias (*p* = 0.09).

**Fig. 12:**
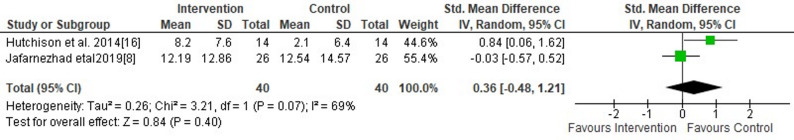
Forest plot illustrating the short-term effects of intervention [total short-term effects of wearing footwear with motion control features (both commercially available motion control footwear without additional modifications and non-commercially available footwear incorporating self-manufactured motion control features] and subgroup analysis of commercially available motion control footwear without additional modifications versus non-commercially available footwear incorporating self-manufactured motion control features) compared to standard shoes as control on peak knee external rotation angle during running. The total effect was calculated as standardized mean differences (95% CI). SD: Standard deviation; Std: Standardized; CI: Confidence interval. Mature: mature females, Young: young females

The authors of two studies evaluated the short-term effects of wearing F-MCF versus standard shoes on peak knee external rotation angle [3, 12]. Our results did not reveal any significant effects on peak knee external rotation angle when using F-MCF compared to standard shoes (Fig. 12). Egger’s test revealed no indication of publication bias (*p* = 0.06).

#### Hip

**Fig. 13:**
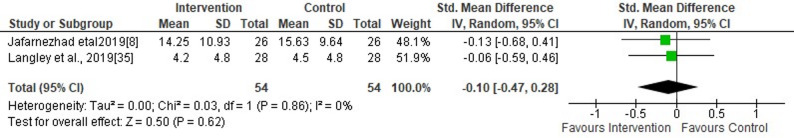
Forest plot illustrating the short-term effects of intervention [total short-term effects of wearing footwear with motion control features (both commercially available motion control footwear without additional modifications and non-commercially available footwear incorporating self-manufactured motion control features] and subgroup analysis of commercially available motion control footwear without additional modifications versus non-commercially available footwear incorporating self-manufactured motion control features) compared to standard shoes as control on peak hip internal rotation angle during running. The total effect was calculated as standardized mean differences (95% CI). SD: Standard deviation; Std: Standardized; CI: Confidence interval

The authors of two studies evaluated the short-term effects of F-MCF versus standard shoes on peak hip internal rotation angle [3, 32]. The findings showed no significant effects of wearing F-MCF versus standard shoes during running on peak hip internal rotation angle (Fig. 13). Egger’s test did not reveal any indication of publication bias (*p* = 0.85).

### Sensitivity Analyses

After removing four studies rated as low quality [4, 29–31], the effects of F-MCF on peak rearfoot eversion remained statistically significant (SMD = −0.94, 95% CI: −1.86 to −0.03, *p* = 0.04, I² = 65%), with similar study heterogeneity (I² = 65%). With regards to rearfoot eversion/inversion excursion, the main result was unchanged after removing studies rated as low quality (Appendix 3: supplementary material). The findings from the sensitivity analyses suggested that the main conclusions are not dependent on low-quality studies and can be considered robust.

Additionally, we computed a heterogeneity-based sensitivity analysis by excluding two studies [28, 29] identified as the main sources of between-study variability. The effect of F-MCFon peak rearfoot eversion remained statistically significant (SMD = −0.45, 95% CI: −0.77 to −0.14, *p* = 0.005, I² = 0%), and study heterogeneity was substantially lower (I² = 0%). With regards to other outcome measures (e.g., rearfoot eversion/inversion excursion and peak ankle eversion), the main results were unchanged after conducting a heterogeneity-based sensitivity analysis (Appendix 3: supplementary material).

Moreover, we calculated a sensitivity analysis excluding one study that explicitly recruited participants with pronated feet [3]. In other words, only findings from studies were aggregated which explicitly reported findings from participants with normal foot posture. The pooled analysis of the remaining five studies showed a significantly lower peak rearfoot eversion angle in the F-MCF condition (SMD = −0.96, 95% CI: −1.58 to −0.33, *p* = 0.003; I² = 70%) (Fig. 2). On average, the peak rearfoot eversion was 3.08° lower (95% CI: −5.28 to −3.18) in the F-MCF condition compared to the control condition. These results support the robustness of our findings.

With respect to peak knee internal rotation, a sensitivity analysis based on foot posture revealed a significantly lower peak knee internal rotation angle in participants presumably having normal feet. We excluded one study that explicitly reported information on participant recruitment with pronated feet [3]. The pooled results from the remaining three studies showed a significant effect favoring F-MCF (SMD = −0.34, 95% CI: −0.67 to −0.01, *p* = 0.05; I² = 1%), indicating a small but consistent reduction in knee internal rotation compared to conventional shoes.

In terms of other outcome measures (e.g., rearfoot eversion/inversion excursion and peak ankle eversion) the main results remained unchanged after conducting a foot posture-based sensitivity analysis (Appendix 3: supplementary material).

## Discussion

In this systematic review with meta-analysis, we aimed to examine the (i) total short-term effects of wearing F-MCF (both CA-MCF and NCA-MCF) versus standard shoes applied during one experimental test session on lower limb joint angles and moments during running in adults and (ii) effects of CA-MCF versus NCA-MCF (subgroup analysis). Based on findings from 14 studies, the meta-analysis revealed that the peak rearfoot eversion angle was 2.85° lower in the F-MCF condition compared to standard shoes with a large effect size of 0.87. Moreover, the peak knee internal rotation angle was 1.42° lower in the F-MCF condition compared to standard shoes with a small effect size of 0.30.

This systematic review with meta-analysis expands upon earlier works [10, 11] by employing stricter inclusion criteria that enhance the clinical relevance of findings. Our analysis also introduces additional kinematic variables (e.g., knee internal rotation), and applies updated quality and sensitivity assessment methods that strengthen the robustness of the conclusions. The observed change in peak rearfoot eversion angle turned out to was statistically significant. Its absolute magnitude (2.85°) exceeds the commonly cited threshold for minimal clinical relevance for rearfoot eversion (2°) [37], suggesting that the effect may be clinically meaningful. The corresponding effect size (SMD = −0.87) is considered large according to Cohen’s thresholds [20], and the direction of the effect was consistent across most included studies. The combination of a statistically significant finding, a large effect size, and an observed rearfoot eversion angle that is above the threshold for being clinically relevant strengthens the potential functional implications of this meta-analysis. These results suggest that even modest reductions in rearfoot eversion through F-MCF may contribute to improve joint mechanics which could ultimately reduce the injury risk, especially in individuals with overpronation.

The results of this study support the idea that when compared to a standard shoe condition, F-MCF (especially CA-MCF) modifications have the potential to induce a reduction in the peak rearfoot eversion angle. Researchers from a previous study [26] have shown that running in a standard shoe compared with F-MCF resulted in significantly higher rearfoot eversion, knee internal rotation angles, and knee external adductor moments. In the study of Lilley et al. [26], F-MCF included an increased sole density and stiffness on the medial side, and a small wedge under the medial heel. Rose et al. [35] suggested that the inclusion of F-MCFconstructions may influence the movement of the foot and orientation of the subtalar axis, which, due to the coupling mechanism between the foot, ankle and tibia, may affect subsequent knee rotation and loading. Cheung and Ng [1] demonstrated that F-MCF (i.e., anti-pronation) reduced the rearfoot eversion angle by 4º in participants with pronated feet and Butler et al. [25] found that CA-MCF significantly reduced tibial and knee internal rotation compared to a standard shoe. However, another study by Stacoff et al. [27], utilising bone pins to directly track the motion of the skeleton, showed that F-MCF (i.e., anti-pronation) did not affect rearfoot motion. These authors suggested that the tibiocalcaneal kinematics of running are unique to each individual and may not be changed by shoe sole modifications. As such, the efficacy of F-MCF elements in running shoes to reduce rearfoot motion requires further investigation. Sensitivity analyses confirmed the robustness of the rearfoot eversion findings. Exclusion of low-quality studies [4, 29–31] preserved statistical significance, while the removal of two studies with large study heterogeneity [28, 29] reduced I² from 66% to 0%. This supports the consistency of the observed effect despite variability in study quality and methods.

The observed difference in peak knee internal rotation angle between F-MCF and standard footwear conditions was small in terms of both absolute angle magnitude (1.42°) and effect size (0.30). This value falls well below commonly reported minimal detectable differences for lower limb joint angles during running (typically 3–6°) [38], raising questions about its functional significance. The corresponding effect size (SMD = −0.30) indicates a small but statistically significant effect according to Cohen’s thresholds [19]. These results suggest a subtle influence of motion control features on proximal joint kinematics, but the magnitude of the effect may be insufficient to infer clinical benefit, and thus should be interpreted within the context of limited functional significance.

The magnitude of rearfoot eversion and subsequent transferal into internal rotation of the knee through tibial rotation has been related to overloading at the knee joint [39]. The kinematic relationship between the rearfoot and knee is complex and cannot be fully described by simple hinge or mitered joint models. Using bone-pin analysis, Stacoff et al. [27] demonstrated that coupling occurs between the shoe and calcaneus, and between the calcaneus and tibia, but that this coupling is phase-specific and highly individualized. For example, coupling between the shoe and calcaneus increased during the unloading phase, whereas calcaneus–tibia coupling decreased, suggesting that inversion and eversion dynamics interact differently with footwear structures depending on the phase of gait and vertical loading [27]. These findings support the notion that F-MCF may influence tibial and knee rotation indirectly, through modulation of rearfoot behavior. However, the large inter-individual variability in coupling responses further emphasizes the need for personalized approaches in footwear prescription and interpretation of biomechanical outcomes. Furthermore, changes in knee joint kinematics were reported in this study, which further highlight the effectiveness of wearing F-MCF to adapt movement patterns higher up in the kinematic chain. Additional work undertaken by the authors of a one study [32] further supports the suggestion that the influence of wearing F-MCF upon joint kinematics reduces as you with movement proximally up the kinematic chain. For example, changes of a larger magnitude were reported for parameters associated with inter-segmental foot kinematics for the same participants running in the same footwear conditions [32].

While this systematic review with meta-analysis confirms previously observed biomechanical changes associated with F-MCF footwear, it also highlights gaps in the literature. One example is, the absence of studies exploring long-term adaptations to sustained footwear use. Real-world shoe usage over several weeks or months may result in neuromuscular adaptations and behavioral changes not captured in single-session studies. Future research should address this by incorporating studies with longitudinal designs, patient-reported outcomes, and functional assessments.

Taken together, these findings suggest that the biomechanical effects of F-MCF are likely context- and individual-dependent. While rearfoot eversion consistently decreases in response to motion control elements, the translation of these changes to proximal joints such as the knee may depend on individual foot structure, gait mechanics, and muscle coordination patterns. This helps explain why some studies report large reductions in tibial or knee rotation, while others show negligible changes.

A suggestion for a future studies is that participants with greater baseline pronation or rotational asymmetry may particularly benefit from F-MCF (e.g., especially CA-MCF) due to a higher margin for correction. Another research question to be addressed in future studies is that neuromuscular adaptations due to the long-term wearing of motion control footwear may mediate how footwear affects kinematic patterns.

### Clinical Implications

The results of this meta-analysis have several potential applications for clinicians who are engaged in injury prevention, gait retraining, and shoe prescription for runners. Most notably, F-MCF (e.g., especially CA-MCF) showed a statistically significant and clinically relevant decrease in peak rearfoot eversion (SMD = −0.87) and a smaller, albeit statistically significant, decrease in knee internal rotation (SMD = −0.30). These results demonstrate that F-MCF is capable of influencing frontal and transverse plane running mechanics and is likely to have an effect on joint loading patterns along the lower limb.

Excessive rearfoot eversion is biomechanically linked to increased tibial internal rotation, leading to aberrant knee mechanics and increased joint stress. These variables are linked to numerous overuse disorders such as patellofemoral pain, medial tibial stress syndrome, and plantar fasciitis [40–42]. By restricting rearfoot movement, F-MCF might therefore assist in the elimination of unwarranted pronation-related movement that is believed to be linked to the development of these disorders.

Although absolute angular reduction in eversion (average difference ~ 2.85°) is lower than traditional minimal detectable differences (3–6°) [38], with large effect size and direction consistency across experiments, there is a probable significant cumulative effect, particularly in susceptible populations.

The noted decrease in knee internal rotation (mean difference approximately 1.42°) is likely clinically relevant, as an increase in tibial rotation is related to patellofemoral joint stress and rotational knee strain [43, 44]. Although small in absolute terms and below established thresholds for clinical detectability, the concurrence of this change with rearfoot motion decreases lends credence to the distal-to-proximal kinematic coupling hypothesis [27, 45].

It should be mentioned that all of the studies involved in this systematic review assessed short-term effects, usually in a single session. Thus, long-term adaptation to F-MCF, such as muscle activation, coordination, comfort, or injury rate changes, has not been researched in this study. Research indicates that biomechanical adaptation to footwear can be different based on individual factors, such as foot posture and arch height [1, 46]. These findings imply that F-MCF are particularly clinically relevant for runners with pronated feet or those returning from injury associated with abnormal rearfoot motion. A targeted, individualistic strategy towards shoe selection, based on biomechanical assessment, will likely yield better outcomes than prescriptions established on general principles [47].

### Limitations

Several included studies did not explicitly report participants’ foot posture. Although we excluded studies that specifically targeted supinated feet or clinical foot conditions, this variability limits the specificity of conclusions regarding individuals with pronated feet. Future studies should report foot posture consistently to allow more refined subgroup analyses. Although we performed sensitivity analyses to control for study heterogeneity and quality, another limitation is heterogeneity arising from the different test protocols applied in the included studies (Table 2). For instance, different motion capture technology, data processing (e.g., sample rate, filter type), and running surfaces can affect kinematic results. This methodological heterogeneity may have caused variance in the meta-analytical findings and needs to be considered when interpreting our results. Standardized reporting and protocol consistency are recommended for future studies. Another limitation of this review is that it does not consider studies that also examined neuromuscular outcomes such as activation patterns of the muscles and coordination, and also neuromuscular control using electromyographic (EMG) data. The integration of these studies could help to better understand the functional impact of the F-MCF on running biomechanics. Future studies should involve EMG analyses and neuromuscular coordination evaluation measures to offer a conclusive picture of how wearing F-MCF affects performance and injury outcomes in running.

### Research Gaps and Future Directions

While the current systematic review synthesized the available evidence on short-term kinematic and kinetic effects of two different types of F-MCF, several important areas remain underexplored. First, only one study assessed neuromuscular parameters such as muscle activation patterns (e.g., via EMG) and another investigated inter-joint coordination strategies, which are critical for understanding the functional responses to footwear interventions. Second, all included studies were limited to acute or short-term effects. No longitudinal studies evaluating physiological adaptations or injury outcomes were available. Third, there were insufficient data to systematically compare different populations (e.g., overpronators vs. neutral runners), limiting generalizability. Future research should aim to integrate kinetic, kinematic, and neuromuscular data and, investigate long-term adaptations according to different motion control footwear types, and account for individual variation in footwear response.

## Conclusions

This study revealed that the wearing of F-MCF versus standard shoes, especially the CA-MCF has the potential to control rearfoot eversion and proximal segment motion in adults. The established effect size and absolute mean difference for the parameter rearfoot eversion angle indicate that our findings are both statistically significant and clinically meaningful. More high-quality research is needed to elucidate the effects of wearing F-MCF on running kinematics and kinetics as well as lower limb muscular activation. This systematic review not only confirms previously observed short-term effects of wearing F-MCF, but also lays the groundwork for more nuanced, individualized, and longitudinal investigations into how footwear design interacts with human movement.

## Supplementary Information

Supplementary Material 1.

## Data Availability

The datasets used and/or analysed during the current study are available from the corresponding author on reasonable request.

## Abbreviations

F-MCF: Footwerar with motion control features
CA-MCF: Commercially available motion control footwear
NCA-MCF: Noncommercially available motion control footwear
CI: Confidence interval
Fig: Figure
HQ: High quality
LQ: Low quality
MQ: Moderate quality
NR: Not reported
PICOS: Population intervention comparator outcomes study design
ROM: Range of motion
SD: Standard deviation
SMD: Standardized mean differences
2D: Two dimensional
3D: Three dimensional
