# Supplementary material for: Short-Term Effects of Wearing Commercially and Non-commercially Available Motion Control Footwear Versus Standard Shoes on Running Biomechanics in Adults: A Systematic Review with Meta-analysis
Source: Sports Med Open. 2025 Nov 21;11:135. doi: 10.1186/s40798-025-00949-z (PMC12638555; doi:10.1186/s40798-025-00949-z)
Supplement: Supplementary file 1 — Supplementary Material 1. [file 40798_2025_949_MOESM1_ESM.docx]

Appendix 1: Search syntax in different electronic databases

Table S1: Search syntax for PubMed from inception until August 2025

| Search number | Query |
| --- | --- |
| 16 | #2 AND #8 AND #14 AND #15 |
| 15 | #11 OR #12 OR #13 |
| 14 | pronation* [tw] OR PRONATED* [tw] OR eversion [tw] OR “motion control” [tw] OR “rearfoot movement” [tw] OR “stable shoe*” [tw] |
| 13 | "Lower Extremity"[Mesh] OR "Lower Extremity Deformities, Congenital"[Mesh] OR "Foot"[Mesh] "Foot Joints"[Mesh] OR "Ankle Joint"[Mesh] OR "Ankle"[Mesh] OR "Knee Joint"[Mesh] OR "Knee"[Mesh] OR "Hip Joint"[Mesh] OR "Hip"[Mesh] OR "Pelvis"[Mesh] OR "Thigh"[Mesh] |
| 12 | "Lower limb*" [ti] OR "lower extremit*" [ti] OR foot [ti] OR feet [ti] OR ankle [ti] OR ankles [ti] OR leg [ti] OR legs [ti] OR knee [ti] OR knee* [ti] OR hip [ti] OR hips [ti] OR pelvis [ti] OR thigh [ti] OR thighs [ti] |
| 11 | "Lower limb*" [tiab] OR "lower extremit*" [tiab] OR foot [tiab] OR feet [tiab] OR ankle [tiab] OR ankles [tiab] OR leg [tiab] OR legs [tiab] OR knee [tiab] OR knee* [tiab] OR hip [tiab] OR hips [tiab] OR pelvis [tiab] OR thigh [tiab] OR thighs [tiab] |
| 9 | #4 AND #8 |
| 8 | #5 OR #6 OR #7 |
| 7 | biomechanic* [ti] OR kinematic* [ti] OR motion* [ti] OR movement* [ti] OR pressure* [ti] OR dynamic [ti] OR load* [ti] OR biomech* [ti] OR mechanic* [ti] OR shock* [ti] OR absorb* [ti] OR friction* [ti] OR moment* [ti] OR angle* [ti] OR rotation* [ti] OR force* [ti] OR "angular impuls*" [ti] OR velocit* [ti] OR speed* [ti] OR acceleration* [ti] OR muscle* [ti] OR activit* [ti] OR torque* [ti] OR power* [ti] OR coordination [ti] OR muscle [ti] OR neuromuscular [ti] OR EMG [ti] |
| 6 | (friction* [tiab] OR moment* [tiab] OR angle* [tiab] OR rotation* [tiab] OR force* [tiab] OR angular* [tiab] Or impuls* [tiab] OR velocit* [tiab] OR speed* [tiab] OR acceleration* [tiab] OR activit* [tiab] OR mechanic* [tiab] OR power* [tiab] OR biomechanic* [tiab] OR kinematic* [tiab] OR motion* [tiab] OR movement* [tiab] OR pressure* [tiab] OR dynamic* [tiab] OR load* [tiab] OR biomech* [tiab] OR mechanic* [tiab] OR shock* [tiab] OR absorb* [tiab] OR coordination [tiab] OR muscle [tiab] OR neuromuscular [tiab] OR EMG [tiab]) |
| 5 | "Biomechanical phenomena"[Mesh] OR "mechanical phenomena"[Mesh] |
| 4 | #1 OR #2 OR #3 |
| 3 | shoe* [tiab] OR footwear* [tiab] OR shod* [tiab] OR “stable shoe*” [tiab] OR “stability shoe*” [tiab] |
| 2 | shoe* [ti] OR footwear* [ti] OR shod* [ti] OR “stable shoe*” [ti] OR “stability shoe*” [ti] |
| 1 | "Shoes" [Mesh] |

Search results based on PubMed 7,891

Table S2: Search syntax for Physiotherapy Evidence Database (PEDro) from inception until August 2025

| First search | Second search | Third search |
| --- | --- | --- |
| • Abstract & title: anterior cruciate ligament* AND | • Abstract & title: shoe** AND | • Abstract & title: footwear* AND |
| • Body part: foot and ankle AND | • Body part: lower leg and knee AND | • Body part: thigh or hip AND |
| • Method: clinical trial | • Method: clinical trial | • Method: clinical trial |

Search results based on PEDro: 379

Table S3: Search syntax for Scopus from inception until August, 2025 (**1,284** results)

| Pronation* [tw] | AND | Shoe* | AND | walk* | AND | biomech* | AND | "lower limb*" |
| --- | --- | --- | --- | --- | --- | --- | --- | --- |
| OR Pronated* [tw] |  | OR Shod* |  | OR walking* |  | OR kinetic* |  | OR "lower extremit*" |
| OR Eversion [tw] |  | OR Footwear* |  | OR gait* |  | OR kinematic* |  | OR foot |
| OR “Motion control” [tw] |  |  |  | OR "translation movement" |  | OR speed |  | OR feet |
| OR “rearfoot movement” [tw] |  |  |  |  |  | OR force* |  | OR ankle |
| OR “stable shoe*” [tw] |  |  |  |  |  | OR motion* |  | OR ankles |
|  |  |  |  |  |  | OR rotation* |  | OR leg |
|  |  |  |  |  |  | OR impuls |  | OR legs |
|  |  |  |  |  |  | OR acceleration |  | OR knee |
|  |  |  |  |  |  | OR dynamic |  | OR knee* |
|  |  |  |  |  |  | OR power |  | OR hip |
|  |  |  |  |  |  | OR movement |  | OR hips |
|  |  |  |  |  |  | OR load* |  | OR pelvis |
|  |  |  |  |  |  | OR joint moment* |  | OR thigh* |
|  |  |  |  |  |  | OR ground reaction force* |  |  |
|  |  |  |  |  |  | OR mechanic* |  |  |
|  |  |  |  |  |  | OR torque* |  |  |
|  |  |  |  |  |  | OR angle* |  |  |
|  |  |  |  |  |  | OR neuromuscular |  |  |
|  |  |  |  |  |  | OR muscle |  |  |
|  |  |  |  |  |  | OR coordination |  |  |
|  |  |  |  |  |  | OR EMG |  |  |

**2,747** results from Scopus

Table S4: Search syntax for Web of Science from inception until August 2025

| Pronation* [tw] | AND | Shoe* | AND | walk* | AND | biomech* | AND | "lower limb*" |
| --- | --- | --- | --- | --- | --- | --- | --- | --- |
| OR Pronated* [tw] |  | OR Shod* |  | OR walking* |  | OR kinetic* |  | OR "lower extremit*" |
| OR Eversion [tw] |  | OR Footwear* |  | OR gait* |  | OR kinematic* |  | OR foot |
| OR “Motion control” [tw] |  |  |  | OR "translation movement" |  | OR speed |  | OR feet |
| OR “Rearfoot movement” [tw] |  |  |  |  |  | OR force* |  | OR ankle |
| OR “stable shoe*” [tw] |  |  |  |  |  | OR motion* |  | OR ankles |
|  |  |  |  |  |  | OR rotation* |  | OR leg |
|  |  |  |  |  |  | OR impuls |  | OR legs |
|  |  |  |  |  |  | OR acceleration |  | OR knee |
|  |  |  |  |  |  | OR dynamic |  | OR knee* |
|  |  |  |  |  |  | OR power |  | OR hip |
|  |  |  |  |  |  | OR movement |  | OR hips |
|  |  |  |  |  |  | OR load* |  | OR pelvis |
|  |  |  |  |  |  | OR joint moment* |  | OR thigh* |
|  |  |  |  |  |  | OR ground reaction force* |  |  |
|  |  |  |  |  |  | OR mechanic* |  |  |
|  |  |  |  |  |  | OR torque* |  |  |
|  |  |  |  |  |  | OR angle* |  |  |
|  |  |  |  |  |  | OR neuromuscular |  |  |
|  |  |  |  |  |  | OR muscle |  |  |
|  |  |  |  |  |  | OR coordination |  |  |
|  |  |  |  |  |  | OR EMG |  |  |

**537** results from Web of Science core collection

Table S5: Search syntax for Cochrane Central Register of Controlled Trials (central) from inception until August 2025

| ID | Search |
| --- | --- |
| #1 | MeSH descriptor: [shoes] explode all trees |
| #2 | MeSH descriptor: [biomechanical phenomena] explode all trees |
| #3 | biomech* |
| #4 | kinetic* |
| #5 | kinematic* |
| #6 | speed |
| #7 | force* |
| #8 | motion* |
| #9 | rotation* |
| #10 | impulse |
| #11 | acceleration |
| #12 | dynamic |
| #13 | power |
| #14 | movement |
| #15 | load* |
| #16 | joint moment* |
| #17 | ground reaction force* |
| #18 | mechanic* |
| #19 | torque* |
| #20 | angle* |
| #21 | neuromuscular |
| #22 | muscle |
| #23 | coordination |
| #24 | EMG |
| #25 | #2 OR #3 OR #4 OR #5 OR #6 OR #7 OR #8 OR #9 OR #10 OR #11 OR #12 OR #13 OR #14 OR #15 OR #16 OR #17 OR #18 OR #19 OR #20 OR #21 OR #22 OR #23 OR #24 |
| #26 | MeSH descriptor: [Lower Extremity] explode all trees |
| #27 | #1 AND #25 AND #26 |

Total of 69 hits for Cochrane Central Register

Appendix 2: Funnel plots

**Figure S1.** Funnel plot indicating potential publication bias for the parameter peak rearfoot eversion angle.

**Figure S2.** Funnel plot indicating potential publication bias for the parameter rearfoot eversion excursion angle.

**Figure S3.** Funnel plot indicating potential publication bias for the parameter rearfoot eversion/inversion excursion.

**Figure S4.** Funnel plot indicating potential publication bias for the parameter peak ankle eversion angle

.

**Figure S5.** Funnel plot indicating potential publication bias for the parameter ankle dorsiflexion/plantarflexion excursion.

**Figure S6.** Funnel plot indicating potential publication bias for the parameter ankle eversion/inversion excursion.

**Figure S7.** Funnel plot indicating potential publication bias for the parameter peak knee flexion.

**Figure S8.** Funnel plot indicating potential publication bias for the parameter knee flexion/extension excursion.

**Figure S9.** Funnel plot indicating potential publication bias for the parameter internal/external rotation excursion.

**Figure S10.** Funnel plot indicating potential publication bias for the parameter peak knee internal rotation angle.

**Figure S11.** Funnel plot indicating potential publication bias for the parameter peak knee external rotation angle.

**Figure S12.** Funnel plot indicating potential publication bias for the parameter peak ankle dorsiflexion/plantarflexion excursion.

Appendix 3: Quality, heterogeneity, and foot posture-based sensitivity analysis

**
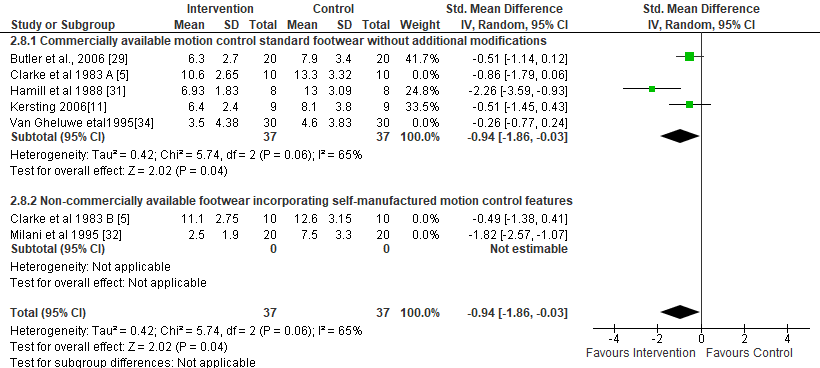
Figure S13.** Quality-based sensitivity analysis for peak rearfoot eversion


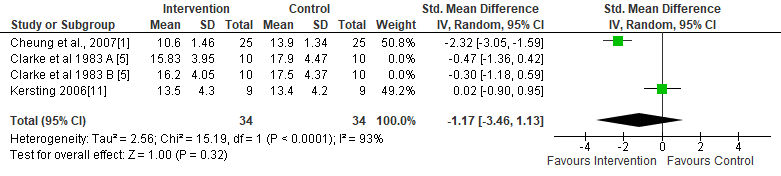
**Figure S14:** Quality-based sensitivity analysis for rearfoot eversion/inversion excursion

**
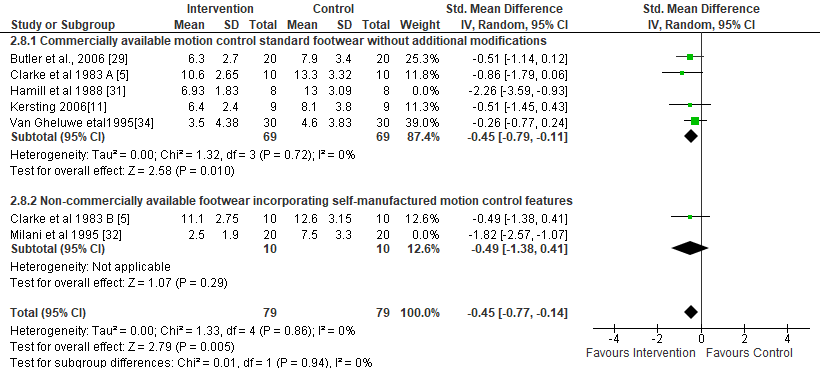
Figure S15:** Heterogeneity-based sensitivity analysis for peak rearfoot eversion


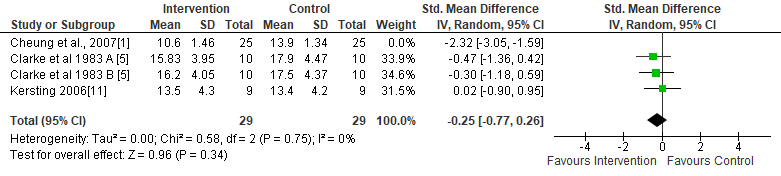
**Figure S16:** Heterogeneity-based sensitivity analysis for rearfoot eversion/inversion excursion


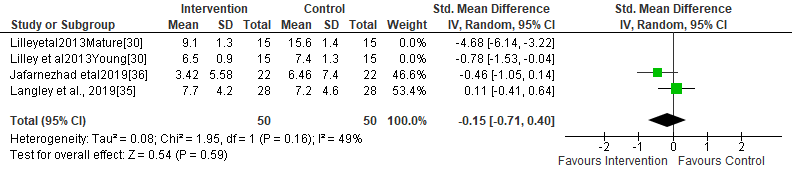
**Figure S17:** Heterogeneity-based sensitivity analysis for peak ankle eversion

**
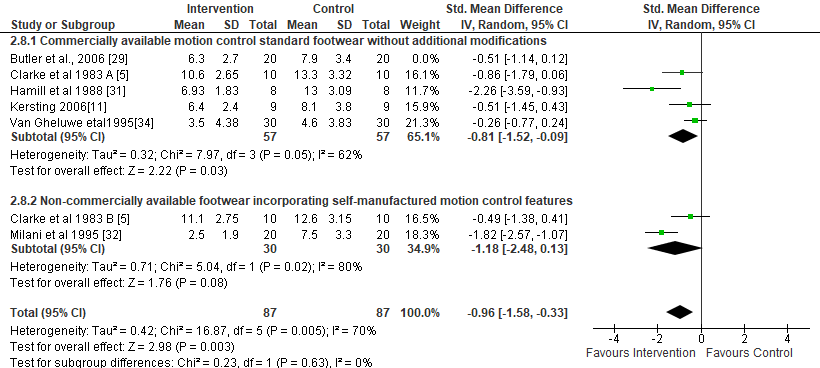
Figure S18:** Foot posture-based sensitivity analysis for peak rearfoot eversion


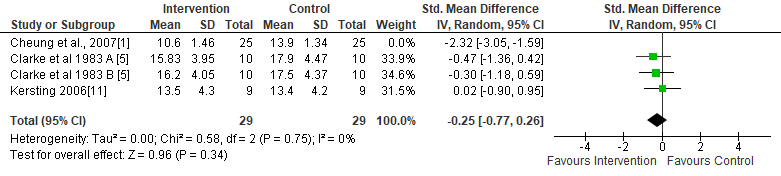
**Figure S19:** Foot posture-based sensitivity analysis for rearfoot eversion/inversion excursion


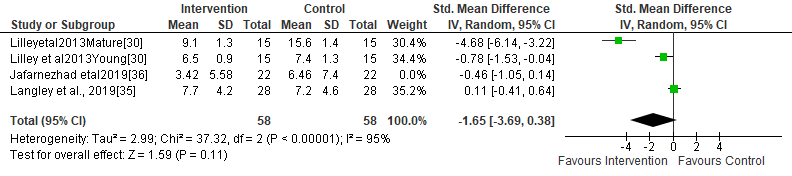
**Figure S20:** Foot posture-based sensitivity analysis for peak ankle eversion


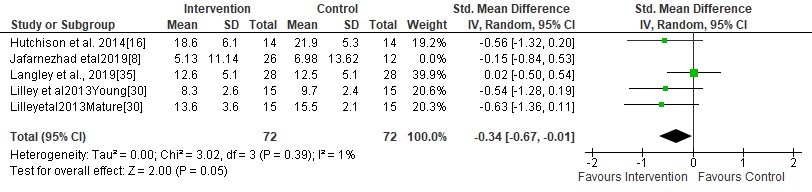
**Figure S21:** Foot posture-based sensitivity analysis for peak knee internal rotation
